# Event inference in multidomain families with phylogenetic reconciliation

**DOI:** 10.1186/1471-2105-16-S14-S8

**Published:** 2015-10-02

**Authors:** Maureen Stolzer, Katherine Siewert, Han Lai, Minli Xu, Dannie Durand

**Affiliations:** 1 Department of Biological Sciences, Carnegie Mellon University, Forbes Ave, Pittsburgh, PA, 15213, USA; 2 School of Medicine, University of Pennsylvania, Curie Blvd, Philadelphia, PA, 19104, USA; 3 Department of Computer Science, Carnegie Mellon University, Forbes Ave, Pittsburgh, PA, 15213, USA

**Keywords:** domains, evolution, phylogenetics, multidomain, reconstruction, domain shuffling

## Abstract

**Background:**

Reconstructing evolution provides valuable insights into the processes of gene evolution and function. However, while there have been great advances in algorithms and software to reconstruct the history of gene families, these tools do not model the domain shuffling events (domain duplication, insertion, transfer, and deletion) that drive the evolution of multidomain protein families. Protein evolution through domain shuffling events allows for rapid exploration of functions by introducing new combinations of existing folds. This powerful mechanism was key to some significant evolutionary innovations, such as multicellularity and the vertebrate immune system. A method for reconstructing this important evolutionary process is urgently needed.

**Results:**

Here, we introduce a novel, event-based framework for studying multidomain evolution by reconciling a domain tree with a gene tree, with additional information provided by the species tree. In the context of this framework, we present the first reconciliation algorithms to infer domain shuffling events, while addressing the challenges inherent in the inference of evolution across three levels of organization.

**Conclusions:**

We apply these methods to the evolution of domains in the Membrane associated Guanylate Kinase family. These case studies reveal a more vivid and detailed evolutionary history than previously provided. Our algorithms have been implemented in software, freely available at http://www.cs.cmu.edu/˜durand/Notung.

## Background

Reconstruction of the history of change in a protein family provides valuable insight into processes of mutation and selection. Evolutionary reconstruction can reveal the context and order in which changes occurred, distinguish between shared history and convergent evolution, and identify interacting mutations that together result in a functional shift. Further, considering protein evolution in the context of species evolution makes it possible to correlate mutations with metabolic, physiological, and morphological changes, indicating which mutations are likely to be functionally important.

Despite tremendous advances in molecular evolution and phylogenetics, methods for reconstructing the evolutionary history of *multidomain *families are lacking. Genes that encode this large and important class of proteins are characterized by a mosaic of sequence fragments that encode structural or functional modules, called *domains*. Multidomain families are central to the two-component histidine kinase signaling systems that are the backbone of cellular communication in prokaryotes. In metazoans, the expansion of multidomain families drove the evolution of cell signaling and cell adhesion. In human health, multidomain families are implicated in tissue repair, apoptosis, inflammation response, antigen recognition, and innate immunity.

**Figure 1:**
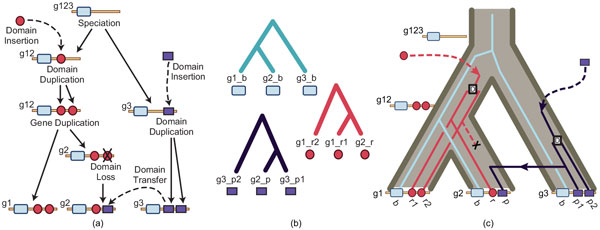
**Schematic of multidomain evolution**. (a) A hypothetical multidomain family evolving by gene duplication and domain shuffling. **(b) **Trees representing the history for each domain in the gene family. **(c) **The evolutionary history of the same family showing the domains evolving in the gene tree. Reconciliation correctly infers 2 domain duplications, 1 domain transfer and 1 domain loss. The gene family (locus) tree is shown in brown; black squares represent domain duplications.

Multidomain families evolve via *domain shuffling *(Figure 1), a process that includes insertion, internal duplication, and deletion of domains. Because gain, loss, or replacement of a domain that encodes specificity can result in an immediate and dramatic change in function, domain shuffling enables rapid evolution of functional variation within gene families that perform core molecular functions.

Understanding the evolution of multidomain protein function requires understanding how domain architectures change over time. Simple domain architecture (DA) models have been used to achieve the computational efficiency necessary for genome-scale analyses [1-4], and work cited therein]. In the DA model, a multidomain sequence is treated as a set or sequence of tokens (e.g. domain names or domain database identifiers [5-10]) representing its domain composition. The DA model has been used to study domain co-occurrence and variation in the domain repertoire across taxonomic lineages [11-13], plasticity in domain order [14,15], domain occurrence graphs [16,17], and domain promiscuity, i.e., the propensity of a domain to co-occur with many other domains [3,18-20].

In a phylogenetic context, patterns of domain gain and loss have been investigated by treating domain architectures as binary character data; a domain is either present or absent in a given architecture. Given a tree with architectures on the leaves, Wagner and Dollo parsimony have been used to infer ancestral domain architectures and the history of domain gains and losses [2,21-24]. Inferring multidomain trees by applying standard phylogenetic methods to domain architectures treated as character data has been proposed, either by calculating the pairwise edit distance between architectures [25] or by employing a parsimony model [21,26]. However, these approaches have not been applied in practical settings. Approaches to infer ancestral states using domain phylogenies have also been proposed [27-29], but these models have resulted in NP-complete optimization problems.

**Figure 2:**
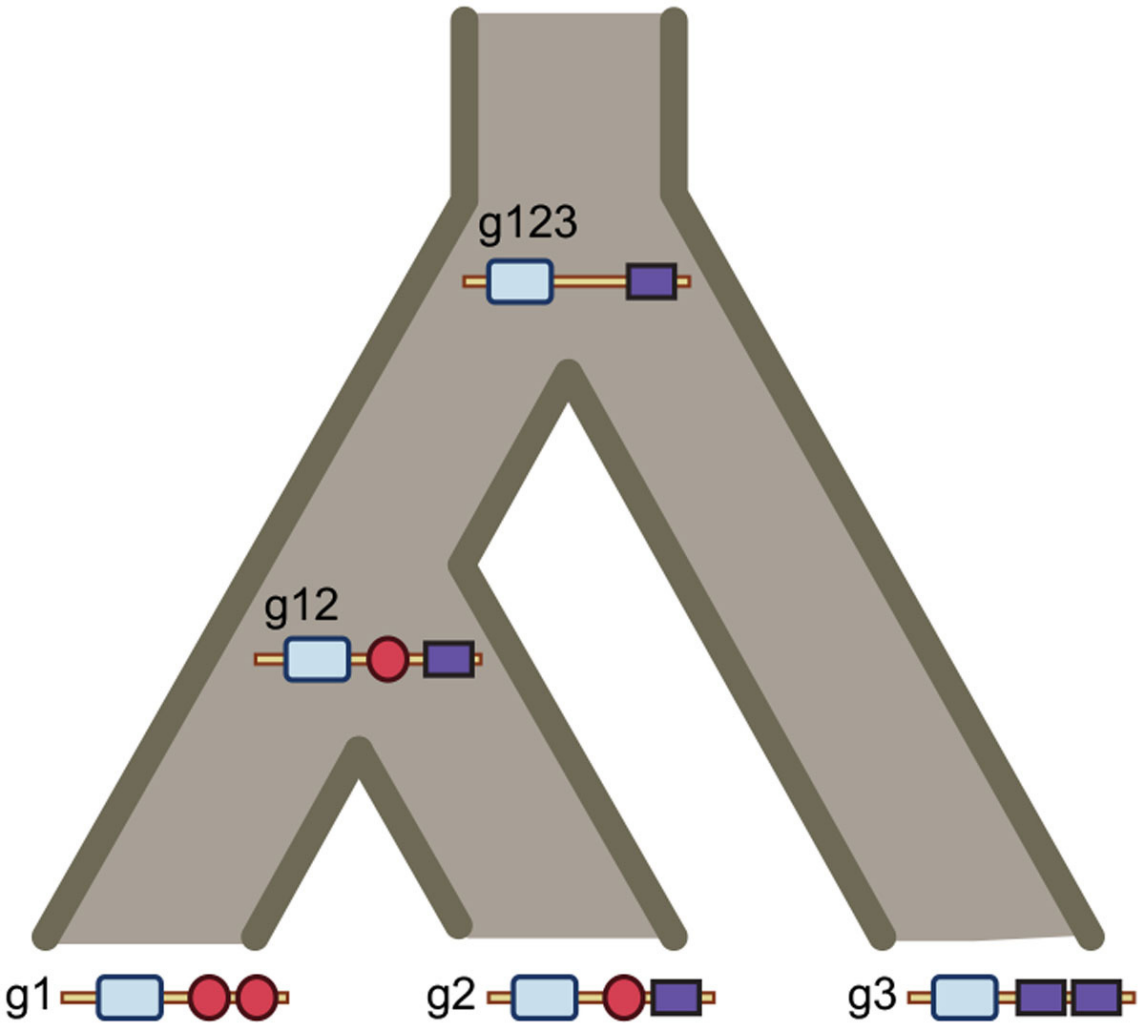
**Domain architecture gain/loss model**. Ancestral domain architectures for the hypothetical family in Fig. 1. Wagner parsimony applied to the the DA model infers 3 gains and 1 loss, underestimating the true events in the "known" history. The ancestral domain architectures inferred with Wagner parsimony are also incorrect.

The benefits of the DA model include computational and conceptual tractability. However, it relies on several unrealistic simplifying assumptions: First, the DA model ignores sequence variation within domain superfamilies, treating all instances of a domain family as indistinguishable. Second, the DA model captures change in the form of domain gain and loss, but is not sufficiently powerful to infer the events that caused the change. For example, a domain gain could result from a domain insertion or an ancestral duplication followed by losses. Without an explicit event model, it is not possible to distinguish between these two scenarios. Third, the DA model will make incorrect inferences and underestimate the degree of domain shuffling activity in the presence of parallel gains or losses, as illustrated in Figure 2.

Here, we introduce a reconciliation-based framework for multidomain event inference. Reconciliation is the process of inferring evolutionary events by comparing the phylogenies of entities at two levels of biological organization. Given a rooted, binary gene tree, a rooted, binary species tree, and a mapping from extant genes to extant species, reconciliation seeks to infer the association between ancestral genes and ancestral species and the optimal history of gene duplications, gene losses, and horizontal gene transfers (HGTs) that explains this association.

Reconciliation for the duplication-loss (*DL*) model was first proposed by Goodman *et al*. [30] and formalized by Page [31] for a parsimony model. Hallett and Lagergren [32] introduced models with transfers and proved that reconciliation under the duplication-transfer (*DT *) model is NP-complete. The field has expanded with further algorithmic development for both the *DL *and *DT *models [33-40], and work cited therein]. Transfers introduce complications that do not occur in a duplication-only model. Transfers introduce degeneracy: there may be more than one optimal combination of transfers, duplications, and losses that gives rise to the same pattern of tree incongruence. Some reconciliation programs generate a single solution, selected at random. More recently, programs that generate all solutions have become available [38,41]. Transfers also introduce temporal constraints: a transfer can only occur between contemporaneous taxa. An event history is only biologically valid if it is *temporally feasible*; that is, if there exists a partial ordering of the nodes in the species tree that satisfies the temporal constraints imposed by the transfers in the inferred event history. There is no known constructive algorithm for generating minimum cost, temporally feasible *DTL*-reconciliations. Instead, dynamic programming is used to construct candidate minimum-cost reconciliations, which are then tested for temporal feasibility. A restricted model that only considers transfers between contemporaneous species, reviewed in [35,40], avoids this problem, but requires a tree with branch lengths in units of time, which are frequently difficult to estimate.

Here we present an event inference algorithm for reconstructing domain evolution. We define a set of domain shuffling events that includes domain duplication, domain loss, domain insertion within the same genome, and horizontal domain transfer between different genomes. We also consider a restricted model that allows domain insertion, but not horizontal transfer of genes or domains. In the context of this model, the events in the history of a multidomain family are inferred by reconciling a domain tree with a gene tree that has been previously reconciled with a species tree. This procedure also yields the timing of those events relative to gene and species divergences, as well as ancestral domain content. Consideration of the co-evolution of domains with both genes and species enables our algorithm to distinguish between domain losses and gene losses, and between domain insertions within the same genome and domain transfers across genomes. Further, our algorithm can determine whether a domain tree co-divergence is due to a species divergence (i.e., a speciation) or a gene divergence (i.e., gene duplication or transfer). In order to ensure the biological validity of the inferred evolutionary histories, we present criteria for temporal feasibility that capture the complex temporal constraints associated with reconciliation involving domains, genes, and species. The implementation of these algorithms is freely available at http://www.cs.cmu.edu/˜durand/Notung. To illustrate the inferential power of our approach, we apply our methods to the Membrane associated guanylate kinase (Maguk) family.

## Results and discussion

### Model

Given a rooted, binary gene tree, *T_GS _*= (*V_G_, E_G_*), a rooted, binary domain tree, *T_D _*= (*V_D_, E_D_*), and a mapping, $M_{L}^{DG}$ from *L*(*T_D_*), the leaves in the domain tree, to *L*(*T_GS_*), the leaves in gene tree, the goal of *domain shuffling event inference *is to infer the association between ancestral taxa in the species, gene, and domain trees and the set of events that best explains this association. In our model, we define the following set of events:

**Co-divergence (***C ***) **A bifurcation in the domain tree that arose through a bifurcation in the gene tree. The gene tree bifurcation may have arisen via speciation (*C_S_*), gene duplication (*C_D_*), or gene transfer (*C_T_*).

**Duplication (D)**A single domain is copied resulting in two separate copies of the domain within the same gene.

**Loss (L)**A domain is deleted from the gene (and genome).

**Domain insertion (***I***) **A new copy of the domain is inserted into a different gene within the same genome.

**Horizontal domain transfer (T)**A new copy of a domain is inserted into a gene in a different genome.

Reconciliation is the process of inferring *M^DG ^*: *V_D _*→ *V_G_*, the association between ancestral domains and ancestral genes. The result is a *reconciled domain tree, T_DG _*= (*V_D_, E_D_*), in which every node, *d *∈ *V_D_*, is annotated with *M^DG^*(*d*) = *g*, where *g *is the ancestral gene that contained domain *d*; $L\;\left(d\right)$, the domains lost on the edge leading to *d*; and E(d), the event that caused the divergence at *d*. Co-divergences and domain duplications correspond to internal nodes in *T_DG_*. Each insertion and transfer corresponds to an edge, (*d*_1_, *d*_2_) ∈ *E_D_*, where *d*_2 _is the inserted (or transferred) domain and *d*_1 _is the donor domain. In a parsimony framework, the cost of a reconciliation is $\kappa =\sum_{\forall \varepsilon }\kappa_{\varepsilon }\cdot n_{\varepsilon }$, where *κ_ε _*is the cost of event ε and *n_ε _*is the number of occurrences of ε in the reconciliation.

Formally, we define the problem of inferring a domain event history as follows:

### Domain Event Inference with Transfers (DE-DTL)

**Domain events: $\left\{C_{S},\;C_{D},\;C_{T},\;D,\;T,\;I,\;L\right\}$**.

**Input: **A rooted, binary domain tree, *T_D _*= (*V_D_, E_D_*); a rooted, binary, *DTL-*reconciled gene tree, *T_GS_*; and a leaf mapping, $M_{L}^{DG}\;:\;L\left(T_{D}\right)\to L\left(T_{GS}\right)$.

**Output: **The set of all temporally feasible, domain shuffling histories *T_DG _*that minimize

$$\kappa =\kappa_{D}n_{D}+\kappa_{T}n_{T}+\kappa_{I}n_{I}+\kappa_{L}n_{L}.$$

Solving DE-DTL entails challenges that do not arise in gene tree-species tree reconciliation. First, when a domain co-divergence is inferred, additional information is required to determine whether the co-divergence is the result of a speciation, a gene duplication, or a gene transfer. Second, domain insertions are horizontal events between genes in the same species. In contrast, domain transfers are horizontal events between different species. An extra test is needed to ensure that the correct event is inferred. Third, a missing taxon in the domain tree may be due to a domain loss or the loss of a gene. If the latter, then the loss should not be included in the event cost of the domain-gene reconciliation. Finally, when testing for temporal feasibility, temporal constraints arising from gene transfers, domain transfers, and domain insertions must all be considered.

*Notation: *Given a tree *T_i _*= (*V_i_, E_i_*), ρ*_i _*designates the root of the tree. For nodes *u, v *∈ *V_i_, p*(*v*) denotes the parent of *v *and *l*(*v*) and *r*(*v*) denote the left child and right child of *v*, respectively. If *u *is on the path from *v *to ρ*_i_*, then *u *is an ancestor of *v*, designated *u *≥*_i _v*, and *v *is a descendant of *u*, designated *v *≤*_i _u*. If $v{≱}_{i}u$ and $u{≱}_{i}v$, then *u *and *v *are *incomparable*, designated $u{≶}_{i}v$. Given a reconciled gene tree *T_GS _*and a reconciled domain tree *T_DG_, s *= *M^GS ^*(*g*) is the species *s *∈ *V_S _*that contained gene *g *∈ *V_G _*and *g *= *M^DG ^*(*d*) is the gene *g *that contained domain *d *∈ *V_D_*. The species containing *d *is *s *= *M^DS ^*(*d*), where *M^DS ^*(*d*) = *M^GS ^*(*M^DG^*(*d*)).

### Domain shuffling with transfers and insertions

Here, we present an algorithm for the domain event inference problem for multidomain families evolving according to a *locus model *[42], in which novel domain arrangements arise through internal duplication, loss, and insertion of domains into an existing gene. This restriction justifies the premises that the history of the family as a whole can be described by a tree. This assumption is consistent with the existence of promiscuous domains that lend themselves to insertion in new chromosomal environments [1,3,43,44] and reports of young genes that arose through duplication of existing genes, followed by acquisition of additional domains [2,45-48]. Moveover, domain insertion into an existing gene is more likely to be viable since all regulatory and termination signals required for successful transcription are present. In addition, we assume that domain insertions and transfers only involve domains within the same gene family. In other words, for a given domain family, we assume that the domain instances that appear in the gene family under consideration form a clade in the domain tree.

In the context of this model, we introduce an algorithm (Alg. 1) for inferring domain events by reconciling a domain tree with a gene tree that has been previously reconciled with a species tree.

**Algorithm 1 **DE-DTL

***Input:*** T_S_; T_GS_; T_D_; $M_{L}^{DG}\;:\;L\left(T_{D}\right)\to L\left(T_{G}\right)$

***Output:*** $\left\{T_{DG}^{1}\ldots T_{DG}^{f}\right\},\;\kappa$

*1 *    $T_{GS}^{*}=addLoss\;\left(T_{GS},\;T_{S}\right)$

*2 *    *costCalc*$\left(\rho_{D},\;T_{GS}^{*},\;T_{S}\right)$

*3 *    $\left\{T_{DG}^{1}\ldots T_{DG}^{m}\right\}=traceback\;\left(\rho_{D},\;T_{GS}^{*},\;T_{S}\right)$

*4 *    $\left\{T_{DG}^{1}\ldots T_{DG}^{f}\right\}=checkFeasibility\;\left(\left\{T_{DG}^{1}\ldots T_{DG}^{m}\right\}\right)$

The algorithm proceeds in four steps. First, an additional data structure, $T_{GS}^{*}$, is constructed. $T_{GS}^{*}$ consists of an extended reconciled gene tree that contains additional nodes and leaves representing taxa that are missing due to gene losses. This additional data structure is used to determine whether or not the donor and recipient of a horizontal event are in the same genome or a different genome, and to distinguish between domain losses and gene losses. Next, candidate reconciliations are generated in two passes over the domain tree. In the first pass, the dynamic program costCalc visits each *d *∈ *V_D _*in post-order and determines the cost of the subtree rooted at *d *for each possible event at *d*. This information is stored in the cost and event tables, K*_d _*and H*_d_*. In the second pass, traceback traverses *T_D _*top-down to generate candidate reconciliations from the information stored in the cost and event tables. In general, there may be more than one optimal set of domain events that reconcile *T_D _*with *T_GS_*. The second pass generates all candidate event histories of minimum cost, $T_{DG}^{1}\ldots T_{DG}^{m}$, where *m *is the number of candidate histories. In the final step, each candidate history is tested for conflicting temporal constraints. The output is the set of all temporally feasible histories, $\left\{T_{DG}^{1}\ldots T_{DG}^{f}\right\}$, where *f *is the number of feasible, optimal reconciliations.

Our domain shuffling event inference algorithm is based on the existing framework for gene-species tree reconciliation with a DTL model [36,49], but contains additional features to address the complications that arise in reconciliation with three nested trees. We discuss these features in detail, here.

addLoss: We construct $T_{GS}^{*}=\left(V_{G}^{*},\;E_{G}^{*}\right)$ from *T_GS _*by placing pseudonodes on each edge (*p*(*g*), *g*) ∈ *E_G_*, on which losses occurred. Each pseudonode represents an ancestral gene that was present in the species lineage from *M^GS ^*(*p*(*g*)) to *M^GS ^*(*g*), but cannot be observed because of a gene loss in one child of each of the intervening species. Let $s_{1}\cdots s_{l}$ be the species between *M^GS ^*(*g*) and *M^GS ^*(*p*(*g*)) that are absent from *T_G _*due to gene losses. We insert pseudonodes ϕ*_s1_*⋯ ϕ*_sl _*between *g *and *p*(*g*) such that ϕ*_s1_*becomes the new parent of *g*, ϕ*_si _*is the parent of ϕ*_si−1_*for *i *= 2 ⋯ *l*, and *p*(*g*) becomes the new parent of ϕ*_sl_*. For each pseudonode, ϕ*_s_*, we attached a pseudoleaf, *λ_s_*, where $s\prime =l\left(s\right)$, if $M^{GS}\left(g\right)≶l\left(s\right)$, and $s\prime =r\left(s\right)$, otherwise. In other words, *S' *is the child of *s *that is not on the path from *M^GS ^*(*g*) to *M^GS ^*(*p*(*g*)) and $\left(s\prime ,\;s\right)$ is the species tree branch on which the loss occurred. Note that a pseudoleaf in $T_{GS}^{*}$ may correspond to an internal node in *T_S_*, because if a gene is missing from an entire clade of species, then the most parsimonious explanation is a single gene loss in the root of the clade.

**Figure 3:**
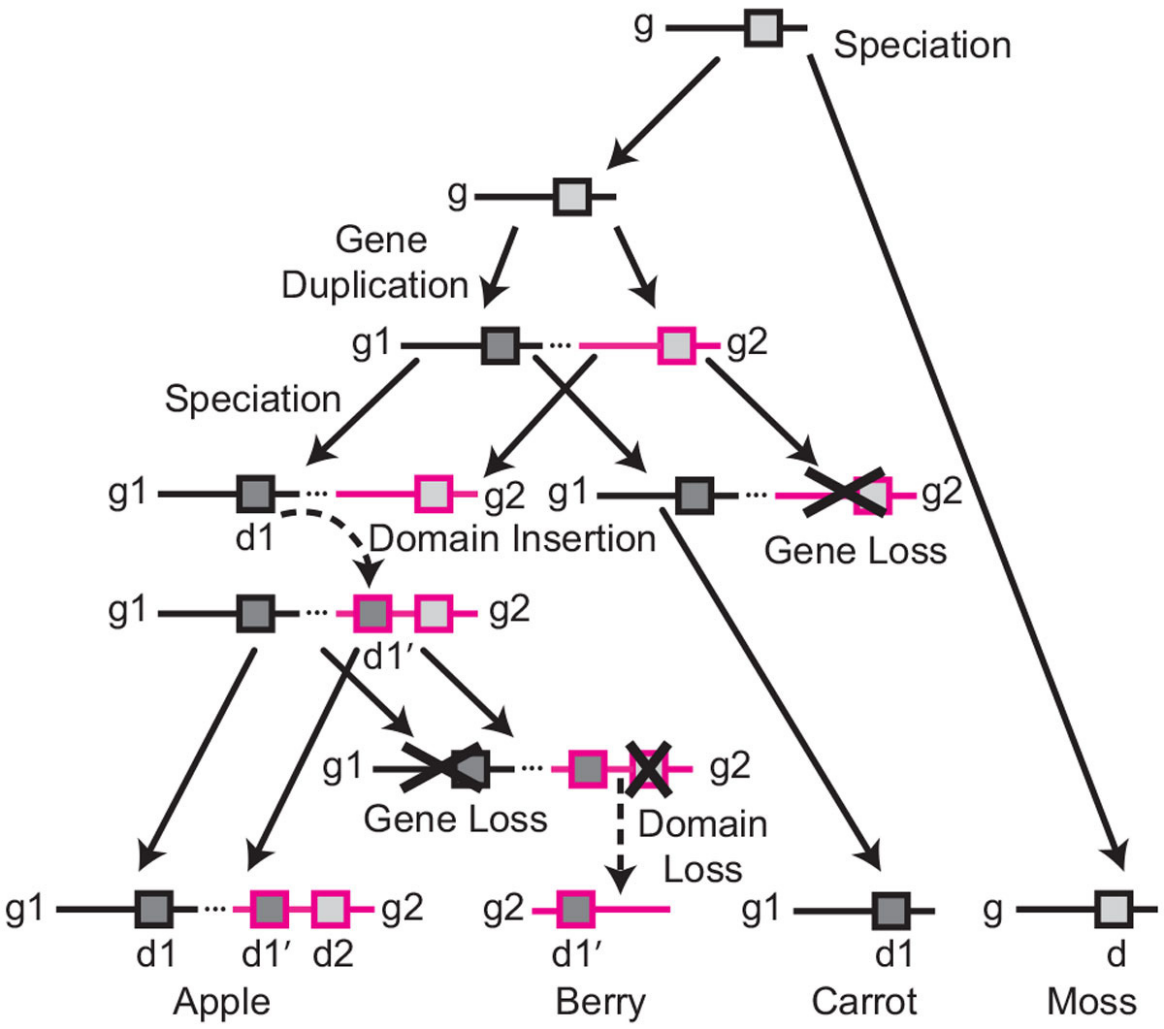
**Evolution of a hypothetical multidomain gene family**. Domain instances are represented by grey squares. Duplicated genes in the same genome are connected by dotted lines.

**Figure 4:**
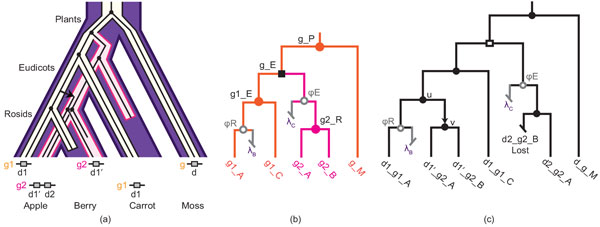
**Domain insertions in the presence of gene loss**. **(a) **Embedded trees showing the co-evolution of domains, genes, and species in the hypothetical family in Fig. 3. **(b) **An extended reconciled gene tree for the gene family. Inferred gene losses (ℓ*_B _*and ℓ*_C _*) and pseudonodes (open circles, ϕ*_R _*and ϕ*_E _*) representing the location of the missing taxa in the gene tree are shown in grey. The pseudonodes are used to distinguish between gene losses and domain losses in the reconciled domain tree. Gene duplication is represented as a black square; filled circles represent co-divergences. **(c) **A reconciled tree for the domain family, showing a domain insertion (arrow, edge (*u, v*)) and a domain loss (*d*2 *g*2 *B*). Domains that are missing due to gene loss (ℓ*_B _*and ℓ*_C_*) are shown in grey. Co-divergence due to gene duplication is represented by an open square.

The addition of pseudonodes allow for more precise estimation of the association between a node in the domain tree and a lineage in the gene tree. Consider, for example, the evolutionary history of the hypothetical family shown in Figure 3, and the reconcilied gene and domain trees for this family, shown in Figure 4. We can infer that the ancestral domain associated with node *u *in *T_D _*was in a genome on the Eudicot-Rosid lineage because of the position of the pseudonode ϕ*_R_*, between *u *and d1_*g*1_*A*. Without the pseudonode, it would not be possible to determine whether or not *u *predates the divergence between Apple and Berry.

Pseudoleaves are also used to distinguish between gene and domain losses. The domain tree in Figure 4(c) has three stubs indicating missing taxa. Two of these are associated with gene losses (ℓ*_B _*and ℓ*_C_*), indicating that only one of the missing domains is due to a domain loss (*d*2_*g*2_*B*).

costCalc: Once the extended reconciled tree has been constructed, the first pass takes *T_D _*and $T_{GS}^{*}$ as input and calls costCalc(*d*) for each *d *∈ *V_D _*in post-order (Alg. 2). The gene association and event at *d *depend on *g_l _*and *g_r_*, the genes associated with the children of *d*. The outer loop in cost Calc enumerates all possible (*g_l_, g_r_*) pairs, and for each pair determines the gene associations and events implied by *M^DG^*(*l*(*d*)) = *g_l _*and *M^DG ^*(*r*(*d*)) = *g_r _*. The cost of each configuration is stored in K*_d_*. A tuple consisting of the event and the mappings of the children of *d *are stored in H*_d_*. The logic for these assignments is as follows.

Domain duplication is the only event that results in children mapped to comparable genes. Thus, if *g_l _*and *g_r _*are comparable, then ε=D and the gene associated with *d *is *g *= *lca*(*g_l_, g_r_*). Horizontal events and co-divergences both result in $g_{l}≶g_{r}$. Therefore, if *g_l _*and *g_r _*are incomparable, both possibilities are considered. For the co-divergence case, the associated gene is again *g *= *lca*(*g_l_, g_r_*). The type of co-divergence is determined by inspecting the gene event associated with gene node *g *in $T_{GS}^{*}$. For the horizontal case, the event is a domain insertion if the donor and recipient gene are in the same genome and a domain transfer if they are in different genomes. For both insertions and transfers, either *g_l _*or *g_r _*may be associated with *d*; i.e., either *g_l _*or *g_r _*could be the donor of the domain.

For each scenario, the cost of domain losses must also be determined, excluding cases where domain loss is due to gene loss. Recall that pseudonodes correspond to gene losses. Therefore, the number of losses, $n_{L}$, on the edge from *d *to *p*(*d*) is the number of non-pseudonodes between *g *= *M^DG^*(*d*) and *g_p _*= *M^DG^*(*p*(*d*)). If *g *and *g_p _*are not pseudonodes, then this quantity is Δ(*g*) − Δ(*g_p_*) − 1, where Δ (*g*) is the depth of *g *in the original gene tree, *T_G_*. However, if *g *is a pseudonode, ϕ, then Δ (*g*) is undefined. We define Δ (ϕ) to be the depth of the first non-pseudonode ancestor of ϕ in $T_{GS}^{*}$. In other words, Δ (ϕ) = Δ (*u*), where *u *≥*_G _*ϕ and there exists no *v *∈ *V_G_*, such that *u *≥*_G _v *≥*_G _*ϕ. This effectively jumps over all pseudonodes between ϕ and *u*. If *g *is a pseudonode and its first non-pseudonode ancestor is a loss, then directly using the depth of the first non-pseudonode ancestor will fail to count this loss. In this case, the number of losses is incremented by one (lines 15 and 16). No such correction is needed when *g_p _*is a pseudonode.

This formulation allows for efficient calculation of the number of losses under each scenario considered in costCalc. If E(*d*) is a co-divergence, then the number of losses is (Δ (*g_l _*) − Δ (*g*) − 1) + (Δ (*g_r_*) − Δ (*g*) − 1). If E(*d*) is a duplication, *g_l_, g_r_*, and *g *should all be at the same depth, and the number of losses is (Δ (*g_l _*) − Δ (*g*)) + (Δ (*g_r_*) − Δ (*g*)).

traceback: Once the first pass is complete, the cost and event tables are filled for each node in *T_D_*. The traceback algorithm constructs a minimum-cost reconciliation using these tables in a pre-order traversal. At every node *d *∈ *V_D_*, the appropriate tuple in the event table H*_d _*is used to assign an event to E(*d*) and determine the labels of the genes associated with the children of *d*. The losses that occurred between *d *and *p*(*d*) are inferred in a climbing procedure [38,50]. For each ancestral node *g *that is missing between *M^DG ^*(*d*) and *M^DG^*(*p*(*d*)), a loss is inferred in *g'*, the child of *g *that is incomparable to *M^DG^*(*d*). If *g *is a pseudonode in *T_GS_*, then *g' *is a gene loss, *λ_s_*. Otherwise, a domain loss in *g' *is inferred.

**Algorithm 2 **DE-DTL: CostCalc

***Input:***$T_{GS}^{*}$; T_D_; $M_{L}^{DG}:L\left(T_{D}\right)\to L\left(T_{G}\right)$

***Output:*** K_d_, $H_{d}\;\forall d\in V_{D}$; ν

*costCalc*(*d*) {

*1 **if **d *∈ *L*(*T_D_*) {

*2    **for each ***$g\in V_{G}^{*}$ {

*3        **if ***$M_{L}^{DG}\left(d\right)≶g$ {*table*(*d, g*, ∞, ∞, *null, null*) }

*4            **else ***{

*5                *$n_{L}=\Delta \left(M_{L}^{DG}\left(d\right)\right)-\Delta \left(g\right)$

*6                table*(*d, g, C_S_,*$n_{L}$*, null, null*)

*7            *}

*8        *}

*9    return*

*10 *}

*11 costCalc*(*l*(*d*))*, costCalc*(*r*(*d*))

*12 ****for each ***$\left(g_{l},\;g_{r}\right)\in V_{G}^{*}\times V_{G}^{*}$ {

*13 g *= *lca*(*g_l_, g_r_*)

*14 *$n_{L}=\Delta \left(g_{l}\right)+\Delta \left(g_{r}\right)-2\cdot \left(\Delta \left(g\right)+1\right)$

*15 ****if***(g_l _is pseudonode) { $n_{L}$ + + }

*16 ****if***(g_r _is pseudonode) { $n_{L}$ + + }

*17 ****if NOT ***$\left(g_{l}≶g_{r}\right)$ {

*18 *    *// Duplication case;*

*19 *    **ε←D**

*20 *    **$n_{L}=n_{L}+2$**

*21 *    *table*(*d, g*, ε, $n_{L}$*, g_l_, g_r_*)

*22 *}

*23 ****else ***{

*24 *    *// Co-divergence case;*

*25 *    ***if $E\left(g\right)=D\left\{\varepsilon \leftarrow C_{D}\right\}$****           // Duplication*

*26 *    ***else if $E\left(g\right)=T\left\{\varepsilon \leftarrow C_{T}\right\}$****   // Transfer*

*27 *    ***else ***{ ε ← *C_S _*}                     *// Speciation*

*28 *    *table*(*d, g*, ε, $n_{L}$*, g_l_, g_r_*)

*29 *    *// Domain insertion or transfer case;*

*30 *    ***if ***M^GS ^(*g_l _*) = *M^GS^*(*g_r_*) {          *// Same genome*

*31 *        *// Domain insertion case;*

*32 *        *table*(*d, g_l_, I*, 0, *g_l_, g_r _*)      *// g_l _to g_r_*

*33 *        *table*(*d, g_r_, I*, 0, *g_l_, g_r _*)     *// g_r _to g_l_*

*34 *    } ***else if $M^{GS}\left(g_{l}\right){≶}_{S}M^{GS}g_{r}$***{

*35 *        *// Domain transfer case;*

*36 *        *table*(*d, g_l_,  T*, 0, *g_l_, g_r _*) *// g_l _to g_r_*

*37 *        *table*(*d, g_r_,  T*, 0, *g_l_, g_r _*) *// g_r _to g_l_*

*38 *    }

*39 *  }

*40 *}

*table*(*d, g*, ε, $n_{L}$*, g_l_, g_r _*) {

*41 ***$\kappa \leftarrow \kappa \left(\varepsilon \right)+K_{l\left(d\right)}\left[g_{l}\right]+K_{r\left(d\right)}\left[g_{r}\right]+\kappa_{L}\cdot n_{L}$**

*42 **if***$\kappa <K_{d}\left[g\right]$ {

*43 *    **$K_{d}\left[g\right]\leftarrow \kappa$**

*44 *    **$H_{d}\left[g\right]\leftarrow \;list\;\left(\left(\varepsilon ,\;g_{l},\;g_{r}\right)\right)$**

*45 *} ***else if***$\kappa =K_{d}\left[g\right]$ { ***list ***($H_{d}\left[g\right]$, (ε, *g_l_, g_r _*)) }

*46 *}

If there is more than one optimal candidate reconciliation, *T_D _*is traversed repeatedly until all minimum cost histories have been generated. The traceback requires no modification to address reconciliation on three levels and is essentially the same as the traceback algorithm for gene tree-species tree reconciliation, as described in [38].

checkFeasibility: Once all candidate reconciliations have been returned, their temporal feasibility is checked to ensure that each solution is valid. This requires criteria for temporal feasibility that accommodate the interplay of domain evolution with gene and species evolution. To be temporally feasible, an inferred domain event history must satisfy two criteria. First, it must be possible to assign a timestamp to every species tree node, such that the timestamps are consistent with the temporal constraints imposed by the combined set of gene and domain transfers. Second, it must be possible to assign a timestamp to every gene tree node, such that the timestamps are consistent with the temporal constraints imposed by the combined set of domain transfers and domain insertions.

For the first criterion, we introduce a *species timing graph*, $G_{t}^{S}=\left(V_{t}^{S},\;E_{t}^{S}\right)$, in which the nodes represent species (i.e., $V_{t}^{S}=V_{S}$) and the edges represent temporal constraints introduced by gene and domain transfers, as well as the temporal relationships imposed by directed tree edges. If gene *g *is horizontally transferred from species *s*_1 _to *s*_2_, then every domain in *g *is also implicitly transferred from *s*_1 _to *s*_2_. Thus, every gene transfer, (*g*_1_, *g*_2_), corresponds to one or more edges in the domain tree. Let $\Lambda_{G}\subset E_{G}^{*}$ and Λ*_D _*⊂ *E_D _*be gene and domain transfer edges in $T_{GS}^{*}$ and *T_DG_*, respectively, and let $\Lambda_{G}^{-}$ be the set of edges (*d*_1_, *d*_2_), such that ∃(*g*_1_, *g*_2_) ∈ Λ*_G_*, where *g*_1 _= *M^DG^*(*d*_1_), *g*_2 _= *M^DG^*(*d*_2_). Then, $\Lambda_{G}^{-1}\cup \Lambda_{D}$ represents all the temporal constraints arising from horizontal transfers of both genes and domains.

The temporal constraints on species are encoded in $G_{t}^{S}$ by adding edges to $E_{t}^{S}$ that represent the following three temporal constraints:

1 If species *s_i _*= *p*(*s_j_*), then *s_i _*must have predated *s_j_*. ∀(*s_i_, s_j_*) ∈ *E_S_*, add (*s_i_, s_j_*) to $E_{t}^{S}$.

2 If a transfer from species *s_i _*to species *s_j _*occurred, then *s_i _*and *s_j _*must have co-existed. Further, the parent of *s_j _*must have predated *s_i_*, and vice versa. $\forall \left(d_{i},\;d_{j}\right)\in \Lambda_{G}^{-1}\cup \Lambda_{D}$, add (*p*(*s_i_*), *s_j_*) and (*p*(*s_j_*), *s_i_*) to $E_{t}^{S}$, where *s_i _*= *M^DS^*(*d_i_*) and *s_j _*= *M^DS^*(*d_j_*).

3 If two transfers occur in the same lineage in the reconciled domain tree (i.e., one is ancestral to the other), then the species corresponding to the donor and recipient of the more ancestral event must have occurred no later than the donor and recipient species of the more recent event. Let (*d*_1_, *d*_2_) and ($d_{1}^{\prime }$, $d_{2}^{\prime }$) be a pair of transfers in $\Lambda_{G}^{-1}\cup \Lambda_{D}$, such that $d_{2}\geq_{D}d_{1}^{\prime }$. Then *s*_1 _= *M^DS^*(*d*_1_) and *s*_2 _= *M^DS^*(*d*_2_) must have occurred no later than species $s_{1}^{\prime }=M^{DS}\left(d_{1}^{\prime }\right)$ and $s_{2}^{\prime }=M^{DS}\left(d_{2}^{\prime }\right)$. Add (*p*(*s*_1_), $s_{1}^{\prime }$), (*p*(*s*_1_), $s_{2}^{\prime }$), (*p*(*s*_2_), $s_{1}^{\prime }$), and (*p*(*s*_2_), $s_{2}^{\prime }$) to $E_{t}^{S}$.

Note that the third constraint considers all pairs of comparable transfers. When mapping gene transfer events to the reconciled domain tree, each pair of transfers that is comparable in *T_G _*corresponds to at least one pair of edges in $\Lambda_{G}^{-}$ that is comparable in *T_D_*.

For the second criterion, we construct a *gene timing graph*, $G_{t}^{G}=\left(V_{t}^{G},\;E_{t}^{G}\right)$, in which the nodes represent genes and the edges represent temporal constraints implied by domain transfers, Λ*_D_*, and insertions, ○*_D_*, where ○*_D _*⊂ *E_D _*denotes the set of domain insertions in *T_DG_*. The nodes in $G_{t}^{G}$ are the nodes in $T_{GS}^{*}$, including pseudonodes (i.e., $V_{t}^{G}=V_{G}^{*}$). Edges are added to $E_{t}^{G}$ to represent the following three temporal constraints:

1 If gene *g_i _*= *p*(*g _j_*), then *g_i _*must have predated *g_j_*. $\forall \left(g_{i},\;g_{j}\right)\in E_{G}^{*}$, add (*g_i_, g_j_*) to $E_{t}^{G}$.

2 If a domain was transferred or inserted from gene *g_i _*to *g_j_*, then *g_i _*and *g_j _*must have co-existed. Further, the parent of *g_i _*must have predated *g_j _*and vice versa. ∀(*g_i_, g _j_*) ∈ {(*M^DG^*(*d*_1_), *M^DG^*(*d*_2_)) | (*d*_1_, *d*_2_) ∈ Λ*_D _*∪ ○*_D_*}, add (*p*(*g_i_*), *g_j_*) and (*p*(*g_j_*), *g_i_*) to $E_{t}^{G}$.

3 Let (*d*_1_, *d*_2_) and ($d_{1}^{\prime }$, $d_{2}^{\prime }$) be domain insertions and/or transfers in Λ*_D _*∪ ○*_D_*, such that $d_{2}\geq_{D}d_{1}^{\prime }$. Then *g*_1 _= *M^DG ^*(*d*_1_) and *g*_2 _= *M^DG^*(*d*_2_) must have existed no later than $g_{1}^{\prime }=M^{DG}\left(d_{1}^{\prime }\right)$ and $g_{2}^{\prime }=M^{DG}\left(d_{2}^{\prime }\right)$. Add (*p*(*g*_1_), $g_{1}^{\prime }$), (*p*(*g*_1_), $g_{2}^{\prime }$), (*p*(*g*_2_), $g_{1}^{\prime }$), and (*p*(*g*_2_), $g_{2}^{\prime }$) to $E_{t}^{G}$.

A domain event history is temporally feasible *iff *both timing graphs are acyclic. If either graph contains a cycle, then the candidate history is infeasible and is not reported. A modified topological sorting algorithm in Θ (|*V_t_*| + |*E_t_*|) [51] is used to test for cycles in *G_t_*.

### Complexity

Our algorithm infers domain shuffling events in polynomial time. addLoss constructs $T_{GS}^{*}$ by adding pseudonodes to *T_GS_*. The number of pseudonodes that can be added to the edge above a node in *T_GS _*is at most *h_S_*, where *h_S _*is the height of *T_S_*. Therefore, the complexity of addLoss is $O\left(\left|V_{G}^{*}\right|\right)=O\left(h_{S}|V_{G}|\right)$. costCalc visits each domain node *d *∈ *V_D _*and loops through all pairs of gene nodes $\left(g_{l},\;g_{r}\right)\in V_{G}^{*}\times V_{G}^{*}$. Since the calculations for each combination of *d, g_l_*, and *g_r _*requires only constant time, the total complexity is $O\left(|V_{D}||V_{G}^{*}{|}^{2}\right)=O\left(|V_{D}|h_{S}^{2}|V_{G}{|}^{2}\right)$.

traceback constructs a single candidate solution in a preorder traversal of the domain tree. Looking up the event, the number of losses, and mapping of each node requires constant time. For each node *d *∈ *V_D_*, the losses between *d *and *p*(*d*) are calculated by climbing from *M^DG^*(*d*) to *M^DG^*(*p*(*d*)). This requires $O\left(h_{G}^{*}\right)$ time for each *d*, where $h_{G}^{*}$ is the height of $T_{GS}^{*}$. Therefore, the total complexity for returning a single condidate reconciliation in the second pass is $O\left(h_{G}^{*}|V_{D}|\right)$. Since the number of pseudonodes added to an edge in *T_GS _*is bounded by *h_S _*and there are at most *h_G _*nodes that are not pseudonodes contributing to $h_{G}^{*}$, $h_{G}^{*}\leq h_{S}h_{G}$. As a result, the complexity of the second pass is *O*(*h_S_h_G_*|*V_D_*|).

To determine whether a candidate solution is temporally feasible, checkFeasibility tests both the gene and species timing graphs for cycles, using a topological sorting algorithm that runs in *O*(|*V_t_*| + |*E_t_*|).

For the species timing graph, $V_{t}^{S}=V_{S}$. The number of edges in $E_{t}^{S}$ depends on the three constraints described in the previous section:

1 |*E_S_*| edges are added to $E_{t}^{S}$, one for each species tree edge.

2 Two edges are added to $E_{t}^{S}$ for each transfer in $\Lambda_{G}^{-}\cup \Lambda_{D}$. This results in the addition of at most 2|*E_D_*| edges because $\left|\Lambda_{G}^{-}\cup \Lambda_{D}|<|E_{D}\right|$.

3 Four edges are added to $E_{t}^{S}$ for every pair of comparable transfers in $\Lambda_{G}^{-}\cup \Lambda_{D}$. Since the number of pairs is bounded by |*E_D_*|^2^, the number of added edges is bounded by 4|*E_D_*|^2^.

Combining all three constraints, the complexity of cycle checking in the species timing graph is *O*(|*V_S_*| + |*E_S_*| + |*E_D_*| + |*E_D_*|^2^). Because |*V_S_*| = *O*(|*E_S _*|) and |*E_D_*| ≥ *E_S_*|, |*E_D_*|^2 ^is the dominant term. Therefore, the complexity can be written as *O*(|*E_D_*|^2^).

For the gene timing graph, $V_{t}^{G}=V_{G}^{*}$ and the number of edges in $E_{t}^{G}$ depends on the three previously described constraints. The first constraint contributes $\left|E_{G}^{*}\right|$ edges to $E_{t}^{G}$. In the worst case, the second and third constraints contribute the same number of edges for the gene timing graph as for the species timing graph, that is *O*(|*E_D_*|) and *O*(|*E_D_*|^2^). Thus, the complexity of cycle checking in the gene timing graph is $O\left(|V_{G}^{*}|+|E_{G}^{*}|+|E_{D}|+|E_{D}{|}^{2}\right)$. Recall that $|E_{G}^{*}|=O\left(\left|V_{G}^{*}\right|\right)=O\left(h_{S}|V_{G}|\right)$. Because |*E_D_*| ≥ |*V_G_*| and |*E_D_*| ≥ *h_S_*, this complexity can be written as $O\left(\left|E_{D}^{2}\right|\right)$.

### Domain shuffling with insertions only

The *DE-DTL *model includes horizontal transfer of both genes and domains and is suitable for reconstructing the history of domain shuffling events in species that accept foreign DNA. However, this model is not appropriate for analysis of multidomain families in species that do not participate in genetic exchange with other species. For such families, we also consider domain shuffling inference for the restricted model without transfer events. This model is particularly well-suited to the large and complex multidomain families in vertebrates [12], in which HGT is thought not to occur.

### Domain Event Inference without Transfers (DE-DL)

**Domain events: $\left\{C_{S},\;C_{D},\;D,\;I,\;L\right\}$**.

**Input: **A rooted, binary domain tree, *T_D _*= (*V_D_, E_D_*); a rooted, binary, *DL-*reconciled gene tree, *T_GS_*; and a leaf mapping, $M_{L}^{DG}:L\left(T_{D}\right)\to L\left(T_{G}\right)$

**Output: **The set of all temporally feasible, domain shuffling histories *T_DG _*that minimize

$$\kappa =k_{D}n_{D}+\kappa_{I}n_{I}+\kappa_{L}n_{L}.$$

The overall structure of the algorithm for DE-DL is the same as for DE-DTL, i.e., Alg. 1. However, since transfers are not allowed, the input gene tree must be reconciled with a species tree under the *DL *model, not the *DTL *model. An extended gene tree, $T_{GS}^{*}$, is constructed using the same procedure as before. With minor modifications, Alg 2 generates solutions to the DE-DL problem. In the co-divergence case, line 26 is omitted to exclude co-divergences with gene transfers $\left(C_{T}\right)$ from the event set. The domain transfer case (lines 34-37) is eliminated altogether. The second pass is identical for both problems, with the exception that transfers do not appear in the cost and event tables.

The worst-case time complexity for costCalc is the same as in DE-DTL, but because domain transfers are not considered and domain insertions are only allowed between genes in the same species, the number of mappings that can be considered is greatly restricted. This suggests a faster run-time in practice.

Determining temporal feasibility is a simpler procedure, since only the gene timing graph must be constructed and tested for cycles. The complexity for gene tree timing graph is *O*(|*E_D_*|^2^).

### Case studies

Here we demonstrate the practical application of our approach using several examples from the Membrane-associated guanylate kinase (Maguk) family [52,53].

**Figure 5:**
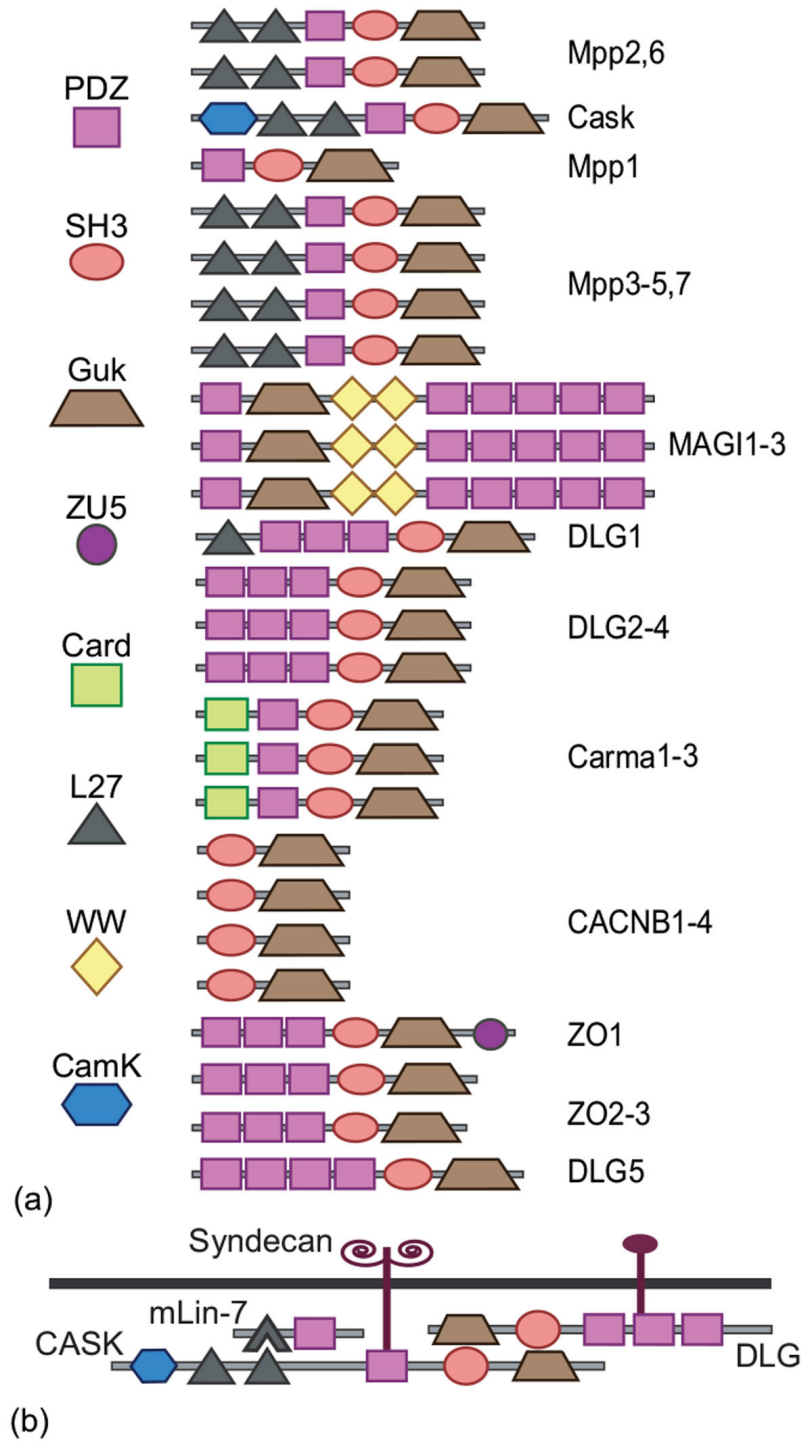
**Multidomain Maguk gene family**. **(a) **Domain architectures found in the Maguk family. Note that *DLG5 *is a member of the *ZO *subfamily [53]. **(b) **Model of a scaffolding complex with two interacting Maguk proteins, adapted from [54].

The Maguks are multidomain scaffolding proteins (Figure 5) that play important roles in cell-cell communication and adhesion including mediating cell polarity [54,55], cell proliferation [56], and synaptic plasticity [57,58]. Scaffolding proteins assemble the components of a signaling cascade in the appropriate configuration [59]. The multidomain architecture is integral to this function because each of the constituent domains is responsible for anchoring specific proteins to the signaling complex. Therefore, acquisition, loss, or replacement of a domain with another from the same family could result in an immediate and dramatic change in interaction partners.

All Maguks have an inactive Guanylate Kinase (*GuK*) domain, in combination with various adapter domains that anchor downstream pathway proteins to the scaffold. There are six Maguk subfamilies, each with a characteristic domain architecture (Figure 5). The identity, copy number, and order of the auxiliary domains is largely conserved within each subfamily, with minor variations.

To analyze the history of domain shuffling in the Maguk family, we require a species tree, a gene tree, and a tree for each domain of interest. For these case studies, we restrict our analysis to three species, human, mouse and chicken, for which the species tree is well known. For the gene tree, we require a phylogeny that represents the history of the entire gene family locus. This can be a challenge for multidomain families with variable domain content, including the Maguks, because it is not possible to obtain a full length sequence alignment, from which to construct a gene tree. In this analysis, we use the phylogeny of the *GuK *domain as a proxy for the history of the gene family. This domain is present in all Maguk architectures and is unique to the Maguk family. In Maguks, the *GuK *and *SH3 *domains participate in intramolecular interactions that cause them to function together as a unit [56,60]. This suggests that the *GuK *domain is under tight structural and functional constraints and, hence, is unlikely to participate in domain shuffling, making it a suitable proxy for the history of the locus.

**Figure 6:**
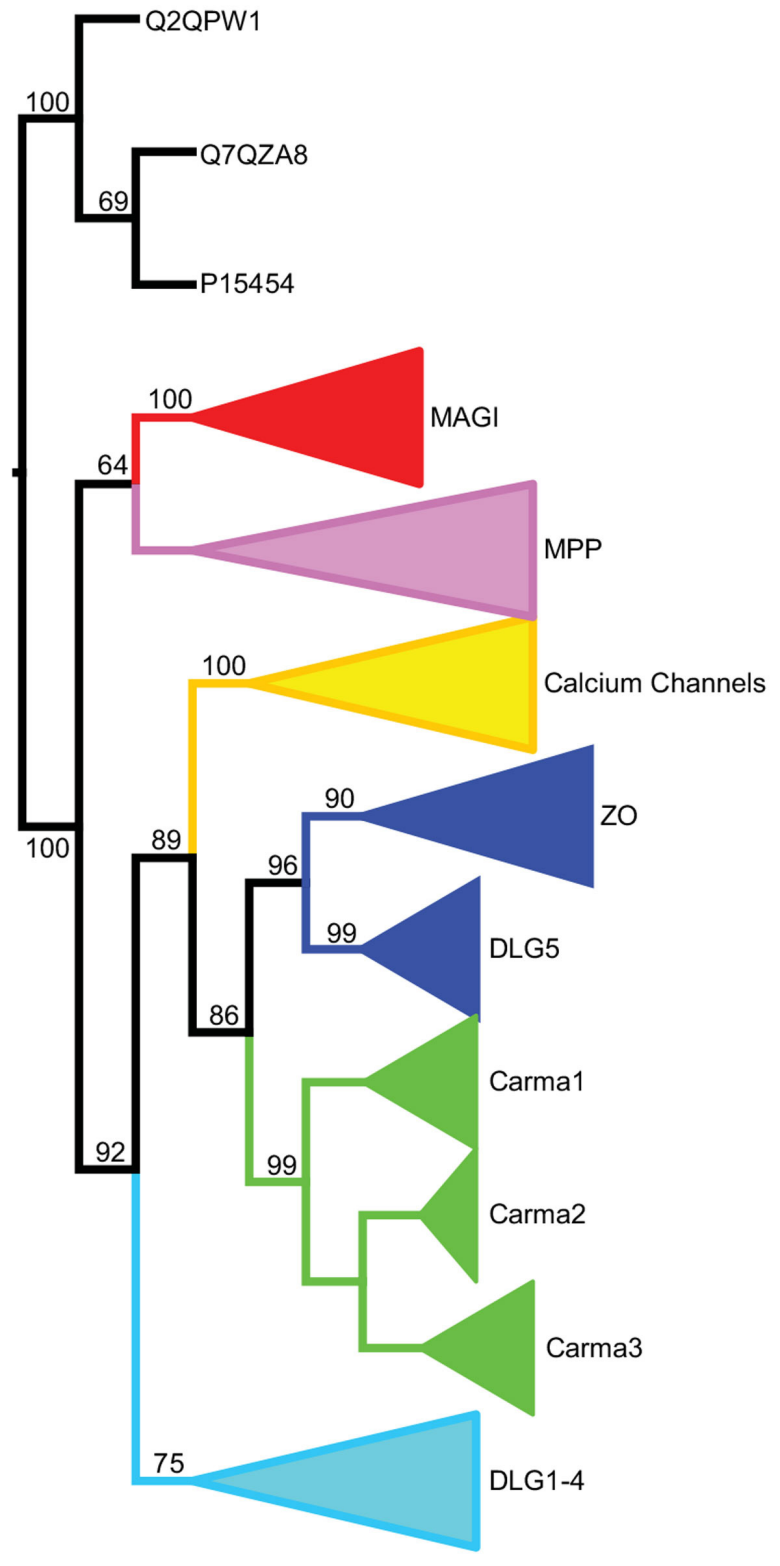
**Phylogenetic relationships of Maguk subfamilies**. Maximum likelihood phylogeny of *GuK *domain sequences. Clades containing paralogous genes from the same subfamily are collapsed. Edge weights are the number of bootstrap replicates, out of 100, supporting that edge.

With this in mind, we constructed a *GuK *domain phylogeny (see Methods) for all members of the Maguk family in mouse, human, and chicken (Figure 6 and Fig. S1 in Additional file 1). Using this gene tree, we investigated possible domain shuffling for two Maguk constituent domains: the *L27 *domain in the Membrane-associated Proteins, Palmitoylated (*MPP*) subfamily, and the *PDZ *domain, which is found in all Maguk subfamilies, except the calcium channel β (*CACNB*) proteins.

*The L27 domain*. The *MPPs *mediate protein complex formation at cell junctions and play a role in establishing cell polarity during development [54]. All *MPPs *contain a core *GuK*-*SH3*-*PDZ *domain architecture and, except *MPP1*, two N-terminal *L27 *domains (Figure 5).

To determine whether the *L27 *domains co-evolved by vertical descent with the Maguk structural core, we constructed trees from the sequences of the first and second *L27 *domains (*L27-1 *and *L27-2*) in human, mouse, and chicken *MPPs *(see Methods). Outside of the *MPP *subfamily, only a few proteins encoded in the human genome possess *L27 *domains (*DLG1*, Lin7A/B/C, MPDZ, INADL). These proteins possess only a single *L27 *domain, rather than a tandem pair. Structural studies partition single-copy and tandem *L27 *domains into two distinct subtypes, with distant sequence homology [61]. This suggests that *MPP L27 *domains are more closely related to each other than to other *L27 *domains and, thus, satisfy the assumption of our algorithm, which only considers domains within the current gene family.

**Figure 7:**
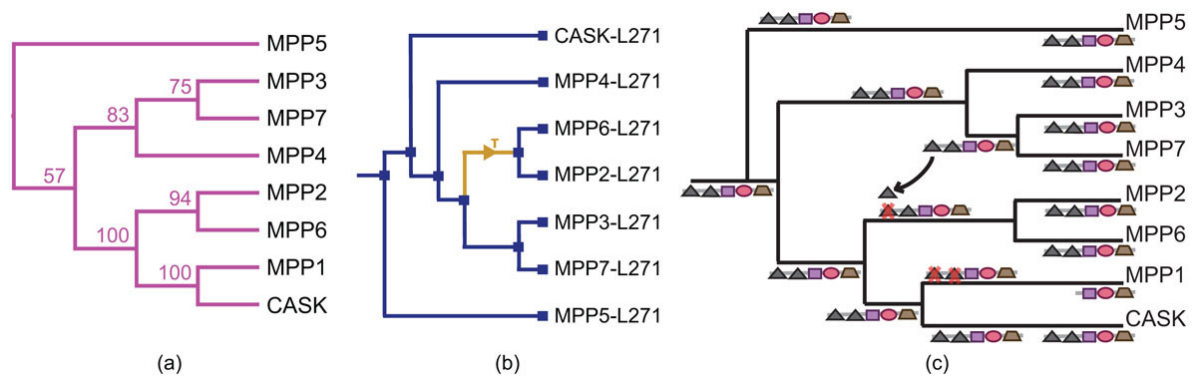
**History of L27 domain shuffling**. **(a) **Phylogeny of the *MPP *subfamily, based on the *GuK *domain maximum likelihood tree. **(b) **The *L27-1 *domain phylogeny showing the inferred domain insertion event. **(c) **Reconstruction of the evolution of the *MPP *domain architecture, showing the replacement of the N-terminal *L27 *domain in the common ancestor of *MPP2 *and *MPP6*. Clades containing mouse, human, and chicken orthologs are collapsed.

The *L27 *phylogeny was reconciled with the *GuK *reference tree (Figure 7(a)) using the DE-DL model and event costs $\kappa_{D}=3$, $\kappa_{I}=1.5$, and $\kappa_{L}=3$. The *L27-2 *subtree (not shown) is consistent the hypothesis that the *L27-2 *and *GuK *domains co-evolved without shuffling. However, the reconciled *L27-1 *tree (Figure 7(b)) suggests that the *L27-1 *domain in the *MPP2/6 *ancestor was replaced by a copy of *L27-1 *from the *MPP3/7 *ancestor, by a single domain insertion (Figure 7(c)).

**Figure 8:**
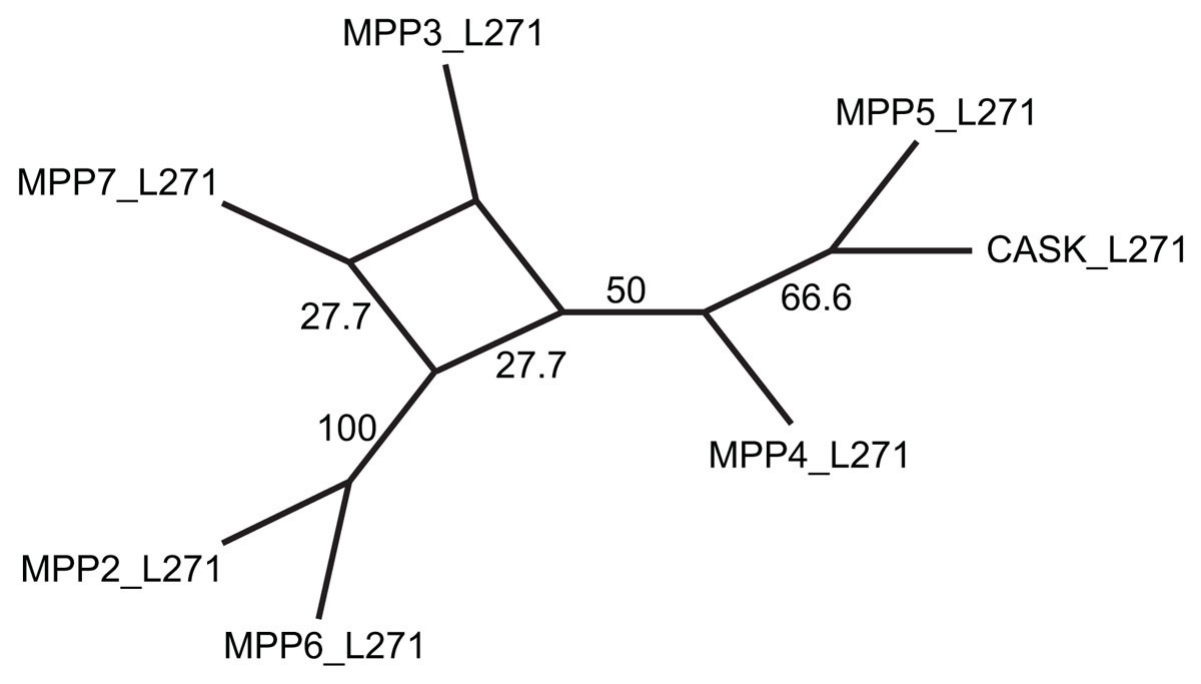
**Consensus network for the L27-1 confidence set**.

Phylogenetic error is of particular concern in domain tree reconstruction, because domain sequences tend to be short and weakly conserved [42]. To investigate the impact of phylogenetic error on the inferred *L27-1 *domain insertion, we generated the 95% confidence set of trees with high likelihood scores [62,63], as described in Methods. Only two topologies are supported by at least 25% of the trees in the confidence set, as shown in the consensus network [64] in Figure 8. In both topologies, *MPP2/6/3/7 *form a clade, which is consistent with the hypothesis that the *L27-1 *domain was replaced in the *MPP2/6 *ancestor.

Previous models of *MPP *evolution [52] predicted that the *L27-1 *domains in *MPP2/6 *and *CASK *share a unique, common ancestor. In contrast, our analysis predicts that the *L27-1 *domains in *MPP2/6 *and *MPP3/7 *are more closely related. This has functional, as well as evolutionary, implications. A vertical descent model implies that *MPP2/6 *are likely to be most functionally similar to *CASK*, their closest *MPP *relative. Our analysis predicts that *MPP2/6 *are more likely to be similar to *MPP3/7*, with respect to *L27 *mediated interactions. This is consistent with recent experimental results that show that *MPP2, MPP3*, and *MPP7 *share a common *L27*-mediated complex formation mechanism, while *CASK *uses a different mechanism [65].

*The PDZ domain*. All members of the Maguk family, except for the *CACNBs*, have at least one *PDZ *domain. Using our newly developed methods, we sought to understand how the varied number of *PDZ *domains arose in this family. Was there a single *PDZ *in the earliest Maguk that expanded independently in different subfamilies? Or was there a round of ancestral domain duplication followed reciprocal, independent losses?

**Figure 9:**
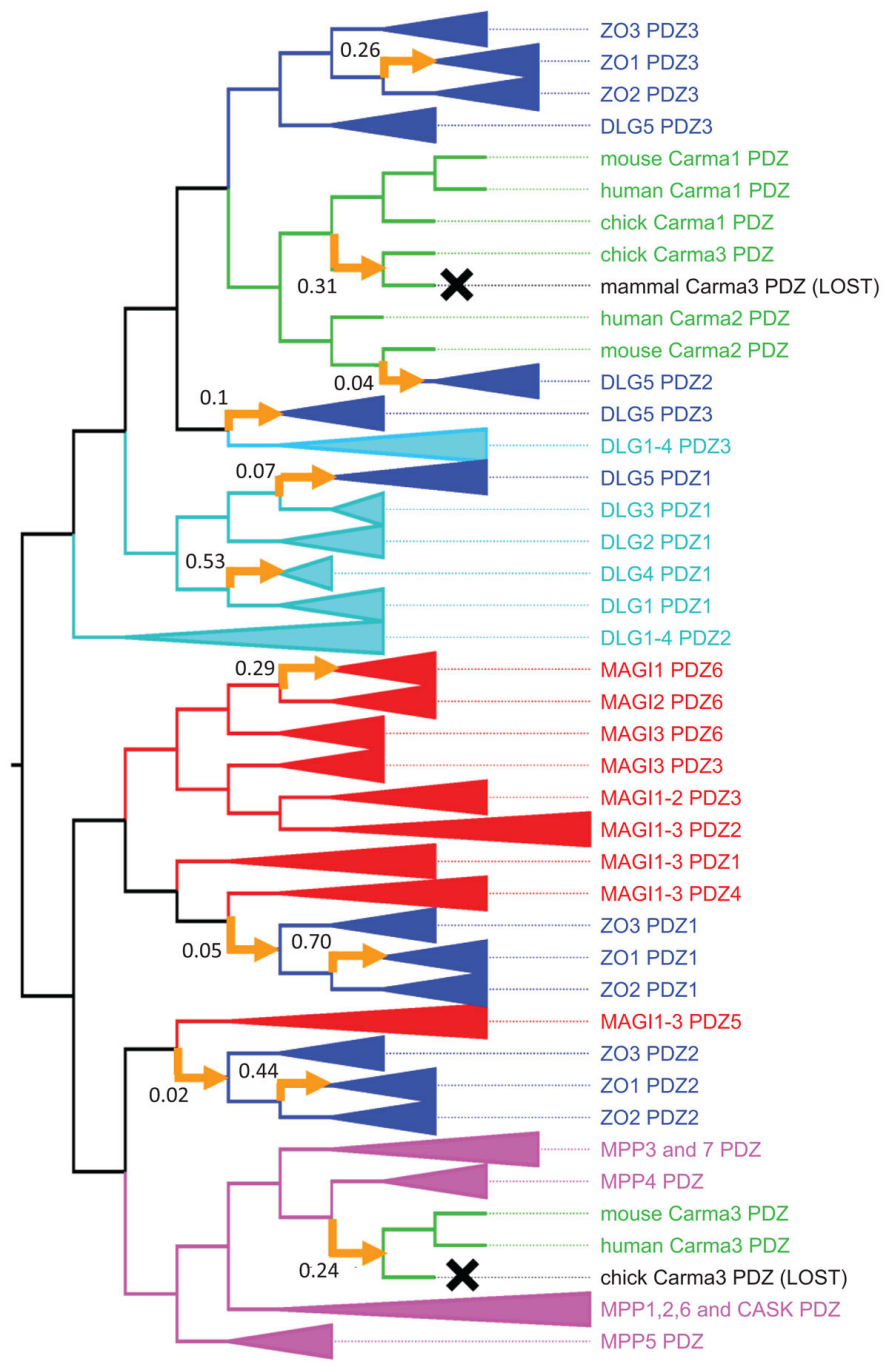
**Maguk PDZ event history**. Based on reconciled domain tree 84, shown in Fig. S2 (in Additional File 1). Leaf labels correspond to protein name, followed by domain name. Domains in each protein are numbered in N- to C-terminal order. Clades containing mouse, human, and chicken orthologs are collapsed, except in the *Carma *family, in which orthologous sequences are not monophyletic. Clades of paralogous genes from the same subfamily are also collapsed. All insertion events that are not within collapsed clades are shown (yellow arrows, annotated with event support values), including seven of the nine high-scoring events. Domain losses are indicated in gray. Gene losses not shown.

The maximum likelihood (ML) tree for the *PDZ *domains (Fig. S2 in Additional file 1) suggests a number of domain shuffling events when reconciled with the *GuK *reference tree. However, many of the relationships in this tree are associated with low bootstrap values and may not reflect an accurate evolutionary history. To investigate the robustness of the inferred domain shuffling events, we generated the 95% confidence set of *PDZ *trees as described in Methods. All 121 trees in this set imply that a number of domain shuffling events occurred - some of these events are unique to a single tree, while others are common to a large fraction of the trees. We defined the support of an inferred event in this set to be the fraction of reconciled *PDZ *trees in which that event occurs (see Methods). The inferred events were scored and ranked based on this support score (Table S1 and Fig. S3 in Additional file 1). The reconciled trees from the *PDZ *confidence set were then ranked based on the number of high-scoring events found in their history. Tree 84 (Fig. S4 in Additional file 1) has nine of the top ten high-scoring insertion events. Only one tree has all top ten events, but that tree results in a much higher reconciliation cost (439.9), compared with a cost of 399.1 for Tree 84. We therefore use Tree 84 in the following analysis of *PDZ *domain evolution in the Maguks (Figure 9).

Our reconstructed history of *PDZ *domain shuffling shows several interesting trends that disagree with prior analyses based on Wagner parsimony. This is especially true for the *ZO *and *Carma *subfamilies.

All members of the *ZO*/*DLG5 *subfamily have a cassette of at least three tandemly arranged *PDZ *domains (and a fourth in *DLG5*). It has been previously been proposed [52] that two domain duplications in the *ZO*/*DLG *ancestor gave rise to this cassette, which was subsequently inherited by all *ZOs *and *DLG5*, with an additional duplication resulting in the fourth *PDZ *in *DLG5*.

**Figure 10:**
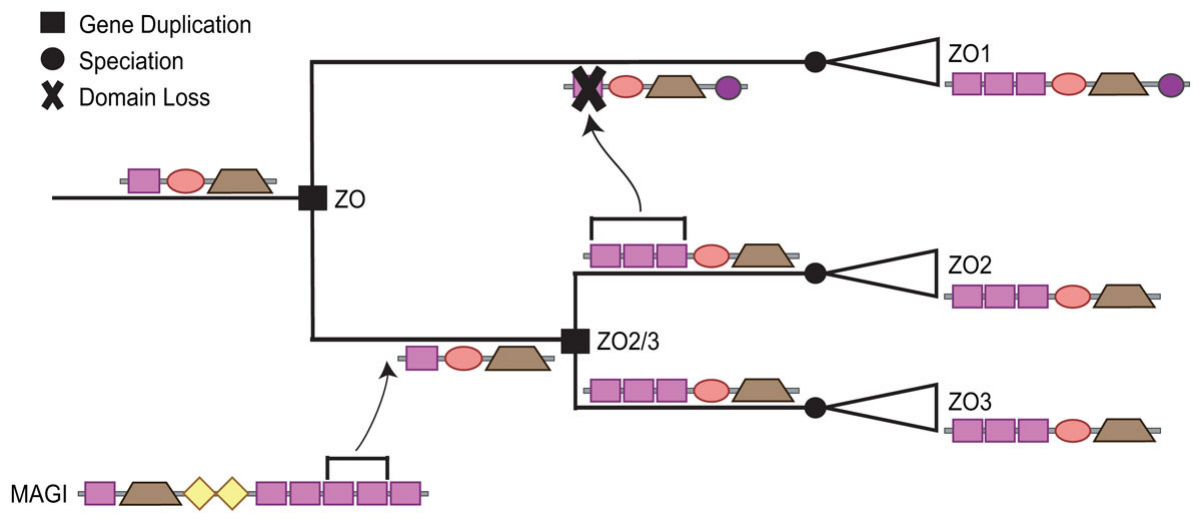
**Reconstruction of PDZ domain shuffling in the ZO subfamily**. Insertion of the two N-terminal *PDZ *domains in the common ancestor of *ZO2 *and *ZO3*, followed by the insertion of the same two domains, plus the ancestral domain, from *ZO2 *to *ZO1*. The ancestral architectures and domain insertions superimposed on the gene tree were reconstructed manually using events and associations of the ancestral nodes in the reconciled domain, gene, and species trees.

Our reconstruction, based on domain tree reconciliation, tells a very different story (Figure 10). First, the *ZO *ancestor had a single *PDZ *domain that was vertically inherited in *ZO2 *and *ZO3*, but lost in *ZO1*. Second, the cassette expansion was the result of a domain insertion from *MAGI *into the common ancestor of *ZO2 *and *ZO3*. While these insertions are weakly supported, the fact that both the donor domains (*PDZ4 *and *PDZ5 *in *MAGI*) and the recipient domains (*PDZ1 *and *PDZ2 *in *ZO2 *and *ZO3*) are adjacent is suggestive. Third, the *PDZ *cassette in *ZO1 *was the result of three, highly supported domain insertion events from *ZO2 *in amniotes. Considering the fact that these three *PDZs *are tandemly located in the domain architecture, it is possible that this was the result of a single insertion event involving all three *PDZ *domains simultaneously. Interestingly, the C-terminal *PDZ *domain in *DLG5 *and the C-terminal *PDZ *in the *ZO *proteins were both vertically inherited from the same ancestral copy. The other *DLG5 PDZ *domains were the result of insertions from the *DLG *and *Carma *subfamilies.

**Figure 11:**
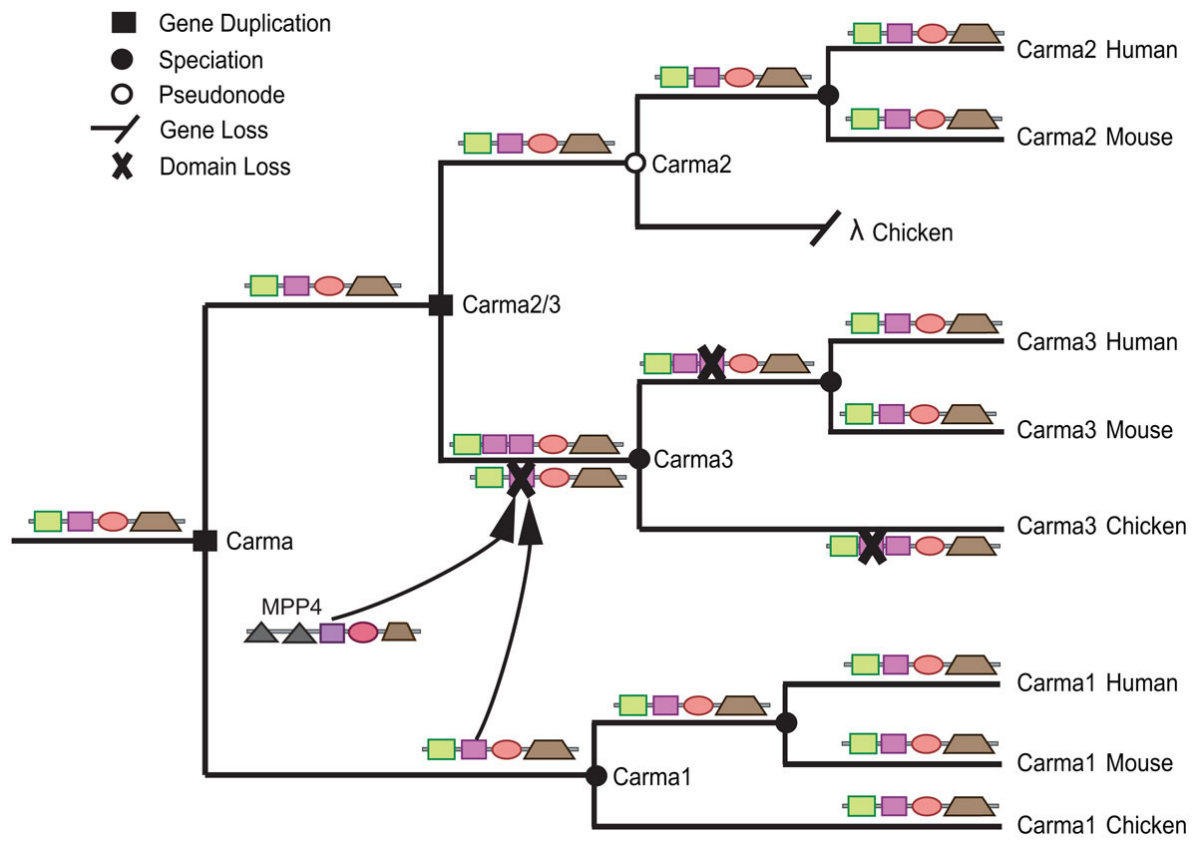
**Reconstruction of PDZ domain shuffling in the Carma subfamily**. Insertion of the *PDZ *domains from *MPP4 *and *Carma1 *into the ancestral *Carma3*. The two copies in *Carma3 *subsequently experienced reciprocal loss - one domain was lost in the mammal ancestor and the other was lost in chicken. *Carma2 *also experienced a gene loss in chicken. The ancestral architectures and domain insertions superimposed on the gene tree were reconstructed manually using events and associations of the ancestral nodes in the reconciled domain, gene, and species trees.

Also of particular interest are the high-scoring insertion events in the *Carma *subfamily. All *Carma *genes have a single *PDZ *domain that is predicted to be the result of vertical inheritance in Wagner parsimony analysis. According to our domain tree reconciliation analysis, however, this is not the case (Figure 11). Although the single *PDZ *domain in *Carma1 *and *Carma2 *was vertically inherited, *Carma3 *experienced both an expansion and contraction of its *PDZ *repertoire in the amniote ancestor. This expansion was due to two independent and highly-supported insertion events - one from *MPP4 *and the other from *Carma1*. Parallel losses followed, with the copy from *MPP4 *lost in birds and the copy from *Carma1 *lost in mammals. As a result, even though all contemporary *Carma3*'s have the same domain architectures, the *PDZ *domain in chicken *Carma3 *is paralogous, not orthologous, to the *PDZ *domain in mouse and human.

These case studies highlight the importance of using information from three levels of evolution - domain, gene, and species - when analyzing the history of a multidomain family. Without multiple species, the divergent history of the *PDZs *in *Carma3 *in birds and mammals would not be apparent. Note also the pseudonode representing the ancestral *Carma2 *in amniotes. This was the result of a loss of the *Carma2 *gene in chicken. Our algorithm correctly identifies the missing *PDZ *in chicken as a gene loss, not a domain loss.

## Conclusions

Here we propose a reconciliation-based framework that captures several aspects of multidomain evolution not represented in the Wagner and Dollo parsimony models that are widelyused in domain architecture analysis. Based on a model that considers domain sequence, as well as domain content, and incorporates an explicit model of events, our reconstruction algorithms are capable of inferring the correspondence between domain architecture evolution and gene and species evolution; the domain duplications, insertions, deletions and transfers that gave rise to present-day proteins; and the ancestral domain architectures from which they evolved.

As a demonstration of the power of a reconciliation-based approach, we presented an analysis of the domain shuffling in the multidomain Maguk family. This analysis uncovered multiple evolutionary scenarios that could not be reconstructed with Wagner parsimony, including parallel gains (the cassette of 3 *PDZs *in the *ZO *subfamily), parallel losses (*PDZ *in *Carma3*), and replacement of a domain with another domain from the same family (*L27 *in the *MPP *subfamily). Further, our results suggest that some orthologous proteins with identical architectures nevertheless contain paralogous domains. This has intriguing implications for orthology-based function prediction.

Our analysis of the Maguks suggests that domain architectures may be much more plastic than previously thought. This contradicts prior studies suggesting that domain architectures rarely form more than once in evolutionary history, based on the argument that domain pairs that co-occur are selectively favorable and once united tend to persist [66]. However, if the same domain architectures were forming repeatedly, this pattern would not be discerned by Wagner parsimony. In contrast, our approach, which incorporates sequence information and an explicit event model, could reveal such patterns. To what extent is our understanding of multidomain evolution driven by the algorithms we use to study them? The results presented here argue for the reexamination of current theories of multidomain protein evolution using more information-rich approaches.

Our empirical study also reveals some of the challenges involved in applying this abstract approach to real data. Reconciliation-based event inference requires accurate domain and gene trees, yet mobile domains tend to be short and have low sequence conservation, making it particularly difficult to infer accurate trees for such domains. In our analysis, we developed an event support score that combines information from multiple phylogenetic hypotheses and focused on events with the strongest support. Development of rigorous approaches for accommodating uncertainty is an essential prerequisite for robust reconciliation-based analyses.

The reconciliation algorithms presented here are the first to consider the co-evolution of domains with both genes and species. A few other studies have considered reconciliation using domain trees for the purpose of inferring domain content in ancestral species [27-29]. The optimization criteria used in those studies do not model domain shuffling events in individual families explicitly. It is therefore unclear whether they can be adapted to the problem of reconstructing the history of events in the evolution of multidomain protein families. Wu *et al*. [67] considered co-evolving domain trees using a model based on gene fusion and fission in a study of domain rearrangements in *Drosophila*. Their approach is inextricably linked to the problem of conserved sequence motif discovery and, similarly, is not well-suited to our question.

Expanding the model presented here is an important area for future work. Our algorithm reconstructs domain events within a single gene family. However, domain shuffling is likely also occurring across gene families. Further, many approaches to domain shuffling, including ours, make the implicit assumption that individual domain events are independent. Algorithms that capture the movement of multiple domains in a single event [68-70] are needed, as are algorithms that take domain order into account. Recent work integrating spatial information with phylogenetic reconciliation [71,72] is a promising direction for reconstructing domain order.

Probabilistic reconciliation models [73-78] are a particularly appealing direction for future work, because they require fewer simplifying assumptions, do not restrict the search space, can be instantiated with different evolutionary models to suit the needs of the data, and provide a context for formal testing of alternate hypotheses. Adapting this approach to the domain architecture context will be challenging. In contrast to gene and protein sequences, which are long strings of symbols from a small alphabet, domain architectures are short sequences from a very large alphabet and may not contain enough information to infer family-specific rates of insertion and deletion.

## Methods

A reference gene tree and two domain trees were constructed for various components of the Maguk gene family. The gene tree was inferred using the non-functional guanylate kinase (*GuK*) domain, which is unique to this gene family. Domain trees were constructed for the two constituent domains that were analyzed in this study: the *L27 *domains of the *MPP *subfamily and the *PDZ *domains, which are present in all Maguks except for the *CACNBs. Sequence acquisition: *An initial set of amino acid sequences of the *GuK, PDZ*, and *L27 *domains from *Gallus gallus, Homo sapiens*, and *Mus musculus *was obtained from te Velthuis *et al*. [52]. This set was verified and expanded using the Uniprot Knowledge Base [79].

*Sequence alignment: *Multiple sequence alignments for the *GuK *and *PDZ *domains were constructed using MUSCLE [80], followed by extensive manual correction. The resulting alignments, with 293 and 107 amino acid positions, respectively, were trimmed in Tri- mAl [81] to remove sites with more than 70% gaps. The final alignments after trimming have 210 and 86 sites, respectively.

*L27 *domain alignments were constructed for the *L27-1 *and *L27-2 *subtypes individually using Expresso [82] under default parameters. Alignments were manually refined based on [61] and then merged using the "combine" function of T-Coffee [83]. These alignments were trimmed manually.

*Phylogeny reconstruction: *Phylogenetic trees for all domain and sequence families were reconstructed using maximum likelihood (ML) estimation. Model selection for all reconstructions was first performed on the trimmed alignment using Modelgenerator [84]. The best model for *PDZ *was LG+G, as supported by AIC1, AIC2, and BIC; the best for *GuK *was JTT+G, as supported by AIC2 and BIC (AIC1 supports JTT+I+G); and the best for *L27 *was JTT.

ML trees for the *GuK, PDZ*, and *L27 *domains were generated in PhyML [85] with 100 bootstraps, based on the selected best models. The *GuK *tree was rooted using the sequences of three functional guanylate kinase domains, from which the *GuK *domain is derived [86]. After rooting the *GuK *tree with these outgroups, one nearest neighbor interchange operation was performed on the least supported branch (bootstrap = 33), so that the *MPP5 GuKs *in all species form a monophyletic clade, instead of grouping some *MPP5 *with *MAGI*. The *L27 *tree was rooted based on the duplication that gave rise to *L27-1 *and *L27-2*.

*TreePuzzling: *Support for the *L27 *and *PDZ *domains was further evaluated by generating a 95% confidence set of trees based on the Expected Likelihood Weight method as implemented in CONSEL [87]. Tree-Puzzle [63] was used to sample 50,000 trees, whose likelihoods were assessed using the JTT model with 4 gamma rate categories. A consensus network was constructed from the *L27 *confidence set using SplitsTree [64] with an edge threshold of 0.25.

*Event support calculation: *For the *PDZs*, the confidence set consists of 121 trees. To avoid spurious event inference, each tree was manually rooted, so as to separate *MPP *and *MAGI *from the other Maguk subfamilies, consistent with the *GuK *tree. Using the DE-DL model, each tree was then reconciled with the reconciled *GuK *tree, as described in the Results and Discussion section, using event costs $\kappa_{D}=3.14$, $\kappa_{I}=8.54$, and 2.72. All multiple optimal solutions were retained for each tree. This resulted in a total of 2358 reconciled trees. Event support was calculated for each inferred domain insertion. The support value reflects the fraction of reconciled trees in which that particular event occurs.

For each insertion observed in at least one reconciliation, we calculate its support as follows: To ensure that each of the 121 trees has equal voting power, the multiple optimal solutions from each tree were averaged. Given a confidence set $\left\{T_{D}^{i}\right\}$ for domain *D*, the support value for insertion ε is

(1) $$\frac{1}{\left|\left\{T_{D}^{i}\right\}\right|}\underset{i}{\sum }\underset{j}{\sum }\frac{I\left(\varepsilon ,\;T_{DG}^{i,j}\right)}{\left|\left\{T_{DG}^{i,j}\right\}\right|},$$

where $\left\{T_{DG}^{i,j}\right\}$ is the set of all optimal reconciliations of $T_{D}^{i}$ and the indicator function $I\left(\varepsilon ,\;T_{DG}^{i,\;j}\right)$ is equal to 1, if $T_{DG}^{i,j}$ contains an insertion that is identical to ε, and is equal to 0, otherwise. Domain insertions (*d*_1_, *d*_2_) and ($d_{1}^{\prime }$, $d_{2}^{\prime }$) in two different reconciled domain trees *T_DG _*and $T_{DG}^{\prime }$ are considered to be identical if these four conditions are satisfied: (1) $L\left(T_{DG}\left(d_{1}\right)\right)=L\left(T_{DG}^{\prime }\left(d_{1}^{\prime }\right)\right)$, (2) $L\left(T_{DG}\left(d_{2}\right)\right)=L\left(T_{DG}^{\prime }\left(d_{2}^{\prime }\right)\right)$, (3) $M^{DG}\left(d_{1}\right)=M^{DG}\left(d_{1}^{\prime }\right)$, and (4) $M^{DG}\left(d_{2}\right)=M^{DG}\left(d_{2}^{\prime }\right)$, where *T_D_*(*d*) is the subtree rooted at *d*.

## Competing interests

The authors declare that they have no competing interests.

## Authors' contributions

The project was conceived and directed by MS and DD. MS, HL, and MX designed and implemented the algorithms. Empirical case studies carried out by KS, MX, and MS. MS, DD, MX, and HL wrote the manuscript. All authors read and approved the manuscript.

## Supplementary Material

Additional file 1 **Additional file containing supplementary figures: stolzer32 supplemental**.pdf Format: PDF Click here for file

## Declarations

Publication charges for this article were funded by NSF grant DBI1262593 to DD.

This article has been published as part of *BMC Bioinformatics *Volume 16 Supplement 14, 2015: Proceedings of the 13th Annual Research in Computational Molecular Biology (RECOMB) Satellite Workshop on Comparative Genomics: Bioinformatics. The full contents of the supplement are available online at http://www.biomedcentral.com/bmcbioinformatics/supplements/16/S14.
