# Supplementary material for: Event inference in multidomain families with phylogenetic reconciliation
Source: BMC Bioinformatics. 2015 Oct 2;16(Suppl 14):S8. doi: 10.1186/1471-2105-16-S14-S8 (PMC4610023; doi:10.1186/1471-2105-16-S14-S8)
Supplement: Additional file 1 — Additional file containing supplementary figures: stolzer32 supplemental.pdf Format: PDF [file 1471-2105-16-S14-S8-S1.pdf]

Supplemental Figures and Tables

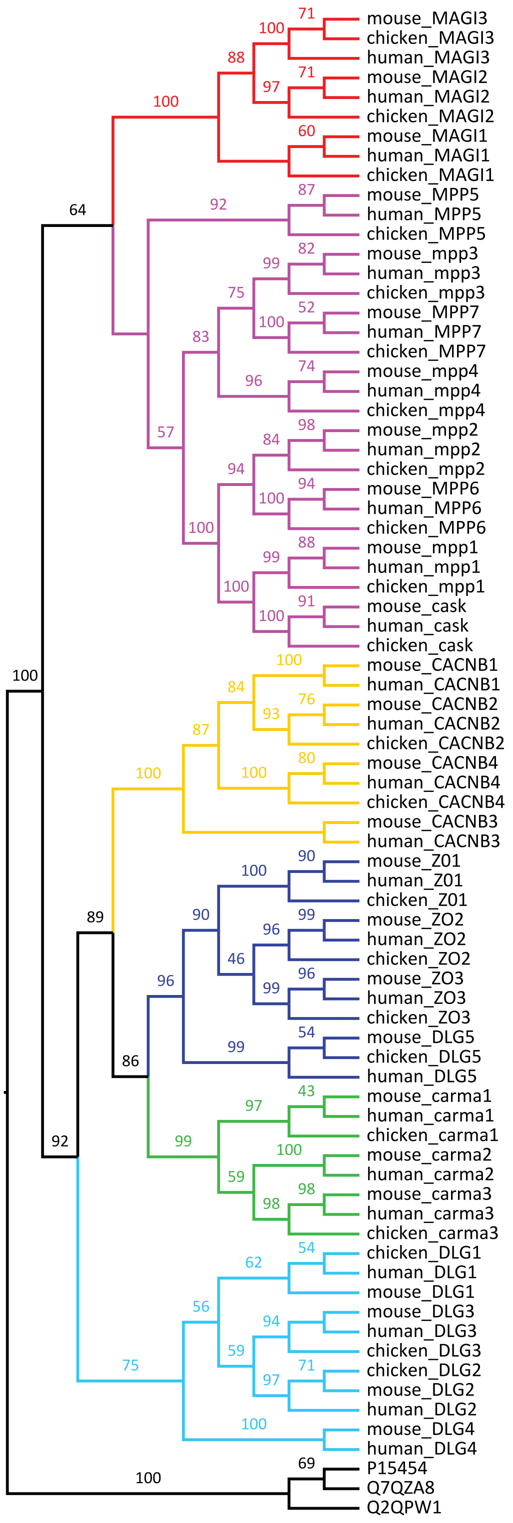

Figure S1 Maguk gene tree. ML phylogeny constructed from the *GuK* domain sequences in human, mouse, and chicken. Edge weights are the number of bootstrap replicates, out of 100, that support each edge.

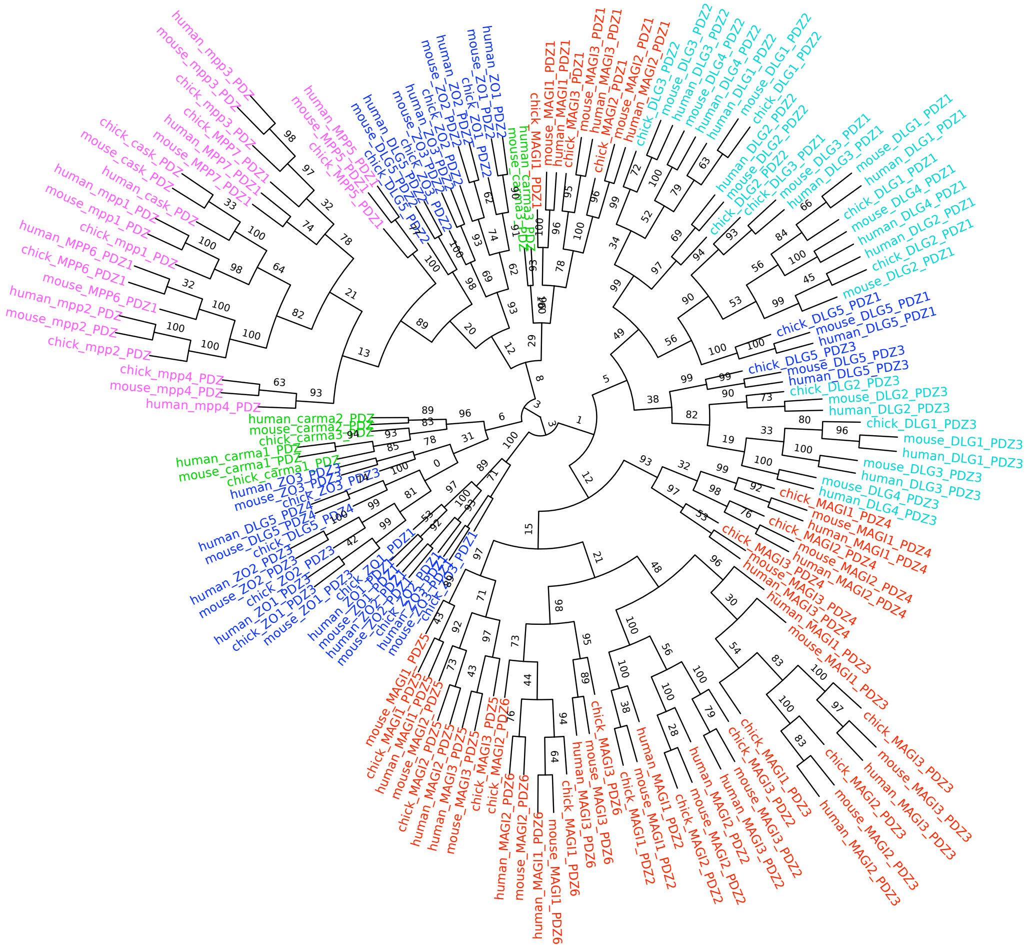

Figure S2 Maguk PDZ domain ML tree in chicken, mouse, and human. Members of the *MAGI* subfamily are shown in red; the *Carma* subfamily in green; the *MPP/CASK* subfamily in purple; the *ZO/DLG5* subfamily in blue; and the *DLG1-4* subfamily in cyan. Bootstrap values, out of 100 replicates, are shown on each edge.

From	To	Support
ZO2	*ZO1*	0.703
DLG1	*DLG4*	0.532
DLG1	*DLG4*	0.441
ZO2	*ZO1*	0.435
Carma1	*Carma3*	0.313
MAGI2	*MAGI1*	0.292
MAGI3	*MAGI1*	0.258
ZO2	*ZO1*	0.255
MPP4	*Carma3*	0.237
DLG1	*DLG4*	0.226

Table S1 Event support for the 10 highest scoring PDZ domain insertions.

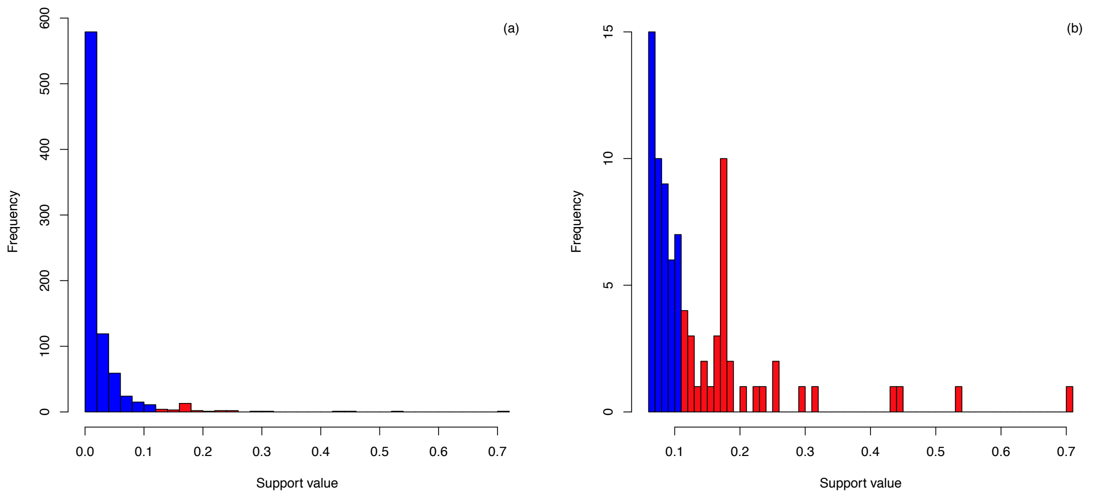

Figure S3 Distribution of event support for PDZ domain insertions. (a) Distribution of support scores for all insertions, with the top 5% shown in red. (b) Distribution of support scores for the top 10% insertions, with the top 5% shown in red.

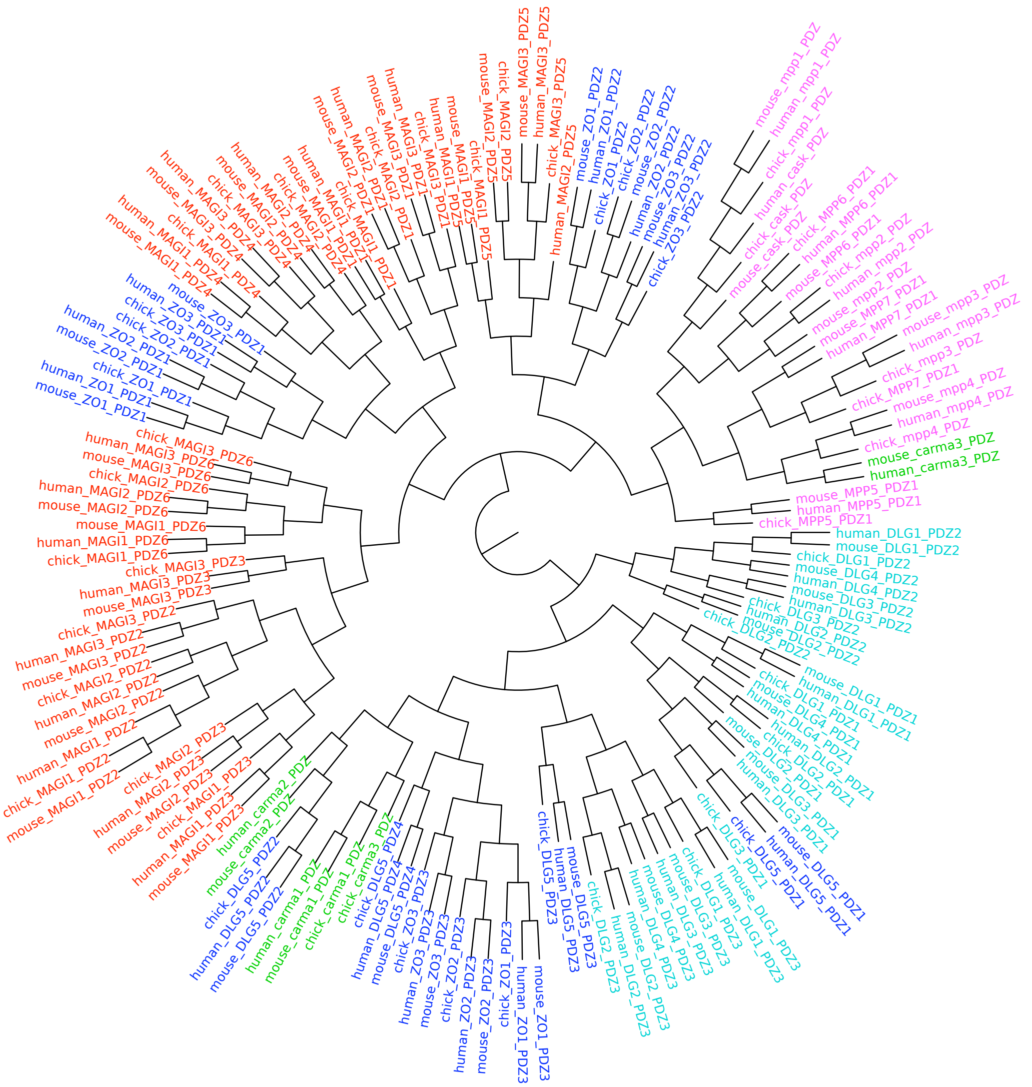

Figure S4 Tree 84 from the confidence set of Maguk PDZ domain trees. Members of the *MAGI* subfamily are shown in red; the *Carma* subfamily in green; the *MPP*/*CASK* subfamily in purple; the *ZO*/*DLG5* subfamily in blue; and the *DLG1-4* subfamily in cyan.
